# Endoscopic endonasal resection of skull base chordoma: case series and review of literature

**Published:** 2012-11

**Authors:** Mohammad Samadian, Seyed Hadi Samimi, Nader Akbari Dilmaghani, Habibollah Moghadasi, Kaveh Ebrahimzadeh, Jahangir Ghorbani, Shahab Khazanehdari

**Affiliations:** ^*a*^Loghman-Hakim Hospital, Shahid Beheshti University of Medical Sciences, Tehran, Iran.; ^*b*^Amiralam Hospital, Tehran University of Medical Sciences, Tehran, Iran.; ^*c*^Day General Hospital, Tehran, Iran.

## Abstract

**Background:**

Skull base chordoma is a rare tumor with slow and progressive growth. Significance of this tumor is it’s difficult to access location in skull base. This is the reason for various proposed techniques for resection of the tumor. Endoscopic endonasal technique is a minimally invasive approach that gives surgeons opportunity of total resection of tumor and low morbidity. Total resection of the tumor is the main determining factor of prognosis.

**Methods:**

In this article we retrospectively studied 18 patients with pathological diagnosis of skull base chordoma treated in Amiralam hospital, Loghman-Hakim hospital and Day general hospital, Tehran, Iran, between 2005 and 2012. All patients underwent endoscopic endonasal surgery. Thirteen patients were primary cases but 3 and 2 cases were referred respectively after radiation failure and recurrence after craniotomy. Mean follow-up time was 23 months.

Difficulty in swallowing and speech, diplopia and nasal obstruction was common presenting symptoms.

**Results:**

Gross tumor resection was feasible in 13 cases. Subtotal resection was done in 5 cases. During follow-up, 1 case died from disease and tumor recurred in other 8 cases. Nine patients are disease free. Eight recurrences and 1 mortality were in cases that underwent subtotal resection or referred to us after radiation failure. The major operative complication was a case of pneumocephalus.

**Conclusions:**

Endoscopic endonasal resection of skull base chordoma is a low morbidity approach, advisable in most cases. We think that total resection is the best surgical strategy. We recommend postoperative radiation in all patients.

**Keywords:**

Endoscopic, Endonasal, Skull base, Chordoma


					Volume 4, Suppl. 1 Nov 2012
Publisher: Kermanshah University of Medical Sciences
URL: http://www.jivresearch.org
Copyright:
Congress of Iranian Neurosurgeons, 14-16 November 2012, Kermanshah, Iran
Paper No. 54
Endoscopic endonasal resection of skull base chordoma: case
series and review of literature
Mohammad Samadian a,*
, Seyed Hadi Samimi b
, Nader Akbari Dilmaghani a
, Habibollah Moghadasi a
,
Kaveh Ebrahimzadeh a
, Jahangir Ghorbani c
, Shahab Khazanehdari a
a
Loghman-Hakim Hospital, Shahid Beheshti University of Medical Sciences, Tehran, Iran.
b
Amiralam Hospital, Tehran University of Medical Sciences, Tehran, Iran.
c
Day General hospital, Tehran, Iran.
Abstract:
Background: Skull base chordoma is a rare tumor with slow and progressive growth. Significance of this tumor is it's
difficult to access location in skull base. This is the reason for various proposed techniques for resection of the tumor.
Endoscopic endonasal technique is a minimally invasive approach that gives surgeons opportunity of total resection of
tumor and low morbidity. Total resection of the tumor is the main determining factor of prognosis.
Methods: In this article we retrospectively studied 18 patients with pathological diagnosis of skull base chordoma
treated in Amiralam hospital, Loghman-Hakim hospital and Day general hospital, Tehran, Iran, between 2005 and
2012. All patients underwent endoscopic endonasal surgery. Thirteen patients were primary cases but 3 and 2 cases
were referred respectively after radiation failure and recurrence after craniotomy. Mean follow-up time was 23
months.
Difficulty in swallowing and speech, diplopia and nasal obstruction was common presenting symptoms.
Results: Gross tumor resection was feasible in 13 cases. Subtotal resection was done in 5 cases. During follow-up, 1
case died from disease and tumor recurred in other 8 cases. Nine patients are disease free. Eight recurrences and 1
mortality were in cases that underwent subtotal resection or referred to us after radiation failure. The major
operative complication was a case of pneumocephalus.
Conclusions: Endoscopic endonasal resection of skull base chordoma is a low morbidity approach, advisable in most
cases. We think that total resection is the best surgical strategy. We recommend postoperative radiation in all
patients.
Key words:
Endoscopic, Endonasal, Skull base, Chordoma
* Corresponding Author at:
Mohammad Samadian: Loghman-Hakim Hospital, Shahid Beheshti University of Medical Sciences, Tehran, Iran. Tel: 09123262849,
Email: mdsamadian@gmail.com, (Samadian M.).

				

